# A Case of Lunacy

**Published:** 1855-10

**Authors:** B. E. Dodson


ARTICLE III.—*A Case of Lunacy*—Ensuing neglect of treatment in the hands of a Homœopathist, successfully treated at Morris, Ill. By B. E. DODSON, M. D.

MRS. E., aged 38, was taken with bilious intermittent fever about the 11th of August—she sent for a Homœopathic doctor. She had at the time, her catamenal period. A few days after the commencement of treatment, a suppression of the discharge took place—a few days after which, a transition of the disease to the brain was manifest. That treatment was continued up to the 19th, during which time the lady was a raving maniac, sometimes contemplating the destruction of her children, &c. As the patie nt was fast sinking from continual incoherent talking, and the ravages of the disease, at the request of the Father of the patient, the

Homœopathic doctor was discharged, and I was called. August 19, 10 o'clock A. M., found the patient with pulse 140, skin cadaverous and cold, extremities cold, tongue coated with dark, brown, heavy coat, patient restless, very nervous—talking all the time, incessantly, and perfectly deranged, with obstinate constipation of the bowels. Administered sulp. morphine 4 grs., applied large blister to the back of the neck, and sinapisms to the hands and feet.

Evening visit found her a little more composed—gave sulp. of morphine 2 grs., ordered the nurse not to allow her to disturb the blister or mustard plasters, which she was disposed to tear off.

20th, 8 o'clock, A. M., patient still delirious, a maniac, had torn off blister and plasters, was perfectly uncontrolable (at this visit my father-in-law, Dr. Edwards, was with me), ordered the hair to be shaved off the head, re-applied blister, covered with tart. emetic, first rubbed the skin with oil of turpentine, tied the patient in straight jacket—gave 80 grs. of sub. murias hydrarg with sulph morphine 3 grs.

Evening visit, no change, gave 60 grs. sub. murias hydrarg, with sulp. of morphine 2 grs.

21st, 8 o'clock, A. M., found patient in a moist sweat, pulse 130, more composed—ordered apperients, composed of salts and senna, and continued until the bowels move.

Evening visit, patient better, would answer some questions, bowels had been evacuated four or five times, skin and tongue moist, pulse 120, gave sulp. of morphine 2 grs., extract of belladonna, ½ gr.

22d, 8 o'clock, P. M., patient improving, pulse 100, called for food, blister very sore and painful, poulticed the blistered surface; ordered gruel, and powders every four hours, composed of sub. murias hydrarg. 2 grs., sulp. of morphine ½ gr., and continued belladonna in ¼ gr. doses, every four hours.

Continued above treatment three days, patient growing more composed and rational, (had taken off straight jacket on the morning of 22d), slight ptyalism induced, gave salts and senna, moved the bowels, gave a few doses of sulp. quinine and opium, during the 25th, 26th, and 27th, and discharged the patient, August 28th, perfectly restored.

Now the question may be asked, why we would continue to give sulp. of morphine in four gr. doses, and 140 grs. sub. murias hydrarg within twelve hours, and continue morphine in two and three grs. doses, &c. In answer I will simply say, that I considered to save my patient from *permanent lunacy*, a prompt work had to be done; and that *potent remedies* were *the means* to be used; and further that I believed the absorbents to be about in the condition that I would expect to find them, in a *continued neglected* case of mania a potu, and I applied my remedies accordingly, and the sequel bears me witness that my diagnosis was correct.

